# Sleep quality and sleepiness in adults with multiple myeloma. Is melatonin a potential treatment?

**DOI:** 10.14814/phy2.70805

**Published:** 2026-03-12

**Authors:** Mariana Fiori, Evellyn M. de Azevedo, Lais S. Rodrigues, Kamily S. Turt, Daniel Pacheco Bruschi, Marcelo M. S. Lima

**Affiliations:** ^1^ Neurophysiology Laboratory, Biological Sciences Sector, Department of Physiology Federal University of Paraná (UFPR) Curitiba Brazil; ^2^ Evolutionary Cytogenomics Laboratory, Department of Genetics, Biological Sciences Sector Federal University of Paraná (UFPR) Curitiba Brazil

**Keywords:** circadian rhythm, melatonin, multiple myeloma, PSQI, sleep quality

## Abstract

Sleep disturbances significantly impact quality of life in cancer patients, yet remain poorly characterized in multiple myeloma (MM). This observational study aimed to characterize sleep profiles in MM patients and evaluate the therapeutic potential of exogenous melatonin. We assessed 46 MM patients and 64 age‐matched controls using the Pittsburgh Sleep Quality Index (PSQI) and Epworth Sleepiness Scale (ESS). Data revealed significantly worse sleep quality in MM patients compared to controls (global PSQI: U = 1126; *p* = 0.034), with 75% of patients exhibiting poor sleep quality or sleep disorders. Component analysis showed specific impairments in sleep latency (*p* = 0.011), sleep duration (*p* = 0.0013), and medication use (*p* = 0.0056). Paradoxically, while MM itself significantly impaired sleep quality, exogenous melatonin supplementation did not ameliorate this effect and was associated with worsened PSQI scores in controls. We observed a dissociation between sleep quality and daytime sleepiness, as ESS scores showed no significant group differences (*p* = 0.58) or treatment effects. These findings provide the first systematic characterization of sleep disturbances in MM, establishing poor sleep quality as a prevalent disease feature. While melatonin influenced specific sleep parameters, its limited efficacy in improving global sleep quality underscores the need for further investigation into standardized chronotherapy approaches for managing sleep–wake disturbances in this population.

## 1 INTRODUCTION

Multiple myeloma (MM) is a malignant hematologic neoplasm, with an incidence in Brazil of 1.24 cases per 100,000 inhabitants, classifying it as a rare disease (Ministério da Saúde, 2022). MM is characterized by the clonal proliferation of plasma cells within the bone marrow, leading to the overproduction of a monoclonal immunoglobulin, known as M protein (Silva et al., 2009). Its pathophysiology results in severe complications, including osteolytic lesions, renal failure, suppressed hematopoiesis, and an increased risk of infection (Gorczyca, 2021). Patients frequently report debilitating symptoms such as bone pain and lesions, maxillofacial manifestations, and persistent fatigue, which can contribute to significant psychological distress and diminished mental health (Rauber & Amâncio, 2023).

Prognosis is commonly assessed using the International Staging System, which categorizes disease severity into stages I (most favorable) to III (least favorable) (Silva et al., 2009). While stage I is most frequently identified at diagnosis in Northern Hemisphere countries, patients in Latin America are often diagnosed at more advanced stages (II or III), a disparity that can further compromise long‐term quality of life (Tietsche De Moraes Hungria et al., 2019). Sleep quality is a critical determinant of overall quality of life in chronic illnesses (Blackwell et al., 2017). However, the impact of MM on sleep remains poorly characterized in the literature, despite the high prevalence of symptoms like pain and fatigue that are known to disrupt sleep architecture. In addition, melatonin, an indoleamine mainly produced by the pineal gland, is known to play a fundamental role in regulating the sleep–wake rhythm. Beyond its chronobiological functions, research over the past 15 years has revealed its involvement in diverse biological processes, including inflammatory, metabolic, and neoplastic pathways (Mogavero et al., 2021).

Considering these dual roles, we hypothesize that sleep disturbances may serve as novel biomarkers for disease monitoring in MM, while melatonin supplementation could offer a promising therapeutic strategy to restore biological rhythms and improve sleep quality. A comprehensive investigation of sleep disturbances and melatonin's therapeutic potential in MM is therefore warranted, as it may not only enhance patient management but also identify novel biomarkers for diagnosis and disease monitoring.

Given this significant gap in the literature, we aimed to comprehensively characterize sleep profiles, particularly sleep quality and daytime sleepiness, in patients with MM and to evaluate the therapeutic potential of exogenous melatonin, a pivotal regulator of circadian rhythms and sleep–wake cycles (Markus et al., 2025; Ribeiro et al., 2025) to improve sleep parameters. This research provides novel insights that could inform clinical strategies to enhance care and quality of life for individuals living with MM.

## 2 MATERIALS AND METHODS

### 2.1 Subjects

This observational study involved a total of 110 volunteers, including 64 individuals in the control group and 46 patients with MM. The control group consisted of caregivers and relatives of the recruited patients, age‐matched to the experimental group. The inclusion criteria were as follows: (i) meeting the diagnostic criteria for MM; (ii) the ability to independently or with assistance from family members complete the questionnaires; Exclusion criteria were as follows: (i) patients who passed by bone marrow transplant; (ii) shift workers; (iii) illiterate participants. The interviews were conducted by the researchers using electronic form allowing the collection of identification data such as name, age, sex, as well as sleep quality parameters and continuous use medications.

### 2.2 Ethics Statement

All experiments were approved by the Ethics Committee under CAAE registration number 73835923.0.3001.0098 and conducted in accordance with the guidelines established by the Ethics Committee for Research with Human Beings of the Erasto Gaertner Hospital, Curitiba, Paraná, Brazil. The study was conducted in accordance with the Declaration of Helsink. All collected data were kept confidential and were accessible only to the principal investigator and the research team to ensure participant privacy. Participants provided written informed consent to take part in the study and for any publication.

### 2.3 Pittsburgh Sleep Quality Index (PSQI)—Brazilian version

The PSQI is a self‐rated questionnaire composed of 19 items organized into 7 different components: subjective sleep quality, sleep latency, sleep duration, habitual sleep efficiency, sleep disturbances, use of sleep medication, and daytime dysfunction. Each component is scored from 0 to 3, resulting in a final score ranging from 0 to 21. A global score of 0–4 indicates good sleep quality; 5–10 indicates poor sleep quality; and a score above 10 is indicative of a sleep disorder (Bertolazi et al., 2011). The PSQI is a tool with good sensitivity and specificity for assessing sleep disorders, and it has been well validated in the literature and has good test–retest reliability (Bertolazi et al., 2011).

### 2.4 Epworth Sleepiness Scale (ESS)—Brazilian version

Daytime sleepiness was evaluated using the Epworth Sleepiness Scale (ESS), a validated self‐report questionnaire that measures an individual's general level of daytime sleepiness (Johns, 1991). The ESS consists of eight items that assess the likelihood of dozing in common daily situations, including: (1) sitting and reading, (2) watching television, (3) sitting inactive in public places, (4) as a passenger in a car, (5) lying down to rest, (6) sitting and talking with someone, (7) sitting quietly after lunch, and (8) while stopped in traffic. For each item, participants rated their probability of dozing on a 4‐point Likert scale (0 = would never doze, 1 = slight chance of dozing, 2 = moderate chance of dozing, 3 = high chance of dozing). Total scores range from 0 to 24, with higher scores indicating greater daytime sleepiness. Consistent with standard clinical interpretation, we considered scores ≥10 as indicative of excessive daytime sleepiness (Johns, 1991, 2010).

### 2.5 Melatonin treatment

Considering this was an observational study in which volunteers undergoing melatonin treatment received varying dosage protocols, ranging from 0.5 to 5 mg/day administered 30–60 min before bedtime. Participants were distributed into two subgroups based on melatonin exposure: a nonexposed group, designated melatonin (−), and an exposed group, designated melatonin (+). For the analysis of the PSQI global score, the groups were as follows: control group melatonin (−) (*n* = 51), control group melatonin (+) (*n* = 10), multiple myeloma (MM) group melatonin (−) (*n* = 33), and multiple myeloma (MM) group melatonin (+) (*n* = 6). For the analysis of the ESS, the groups were: control group melatonin (−) (*n* = 53), control group melatonin (+) (*n* = 11), multiple myeloma (MM) group melatonin (−) (*n* = 35), and multiple myeloma (MM) group melatonin (+) (*n* = 6). Due to the limited sample size, which would have resulted in underpowered statistical comparisons, we did not further stratify the melatonin (+) subgroup by dosage.

### 2.6 Statistical analysis

All datasets were first assessed for normality using the Kolmogorov–Smirnov test, which indicated nonparametric distributions for all variables. Associations between groups (control vs. MM) and categorical variables (sex and age group) were assessed using odds ratios (OR) with 95% confidence intervals, using the control group, female sex, and the youngest age category as reference groups, respectively. Furthermore, a Cochran‐Armitage test for trend was specifically applied to the ordinal age variable to evaluate ordinal categories. Group comparisons were performed using the Mann–Whitney *U* test for two‐group analyses and the Kruskal–Wallis test followed by Wilcoxon post hoc tests with Bonferroni correction for stratified comparisons of melatonin treatment effects. A significance threshold of *p* ≤ 0.05 was maintained for all analyses. Continuous variables are presented as mean ± standard deviation. The analyses were conducted using RStudio (version 2025.05.1+513) and GraphPad Prism (version 8.4.3, GraphPad Software, San Diego, CA, USA).

## 3 RESULTS

### 3.1 Demographic and clinical characteristics

The demographic characteristics of the study participants are summarized in Table 1. The control group comprised 76% female and 24% male volunteers, while the MM group consisted of 72.7% female and 27.3% male volunteers. The odds of being male were 1.17 times higher in the MM group compared to the control group. However, the OR was not significantly different from 1.0 (OR = 1.17, 95% CI [0.49, 2.83], Fisher's Exact test *p* = 1.00), indicating no statistically significant association between sex and volunteer group.

**TABLE 1:** Demographic characterization of the participants.

Parameters	Groups		Values	
	Control	MM	OR	*p*
Sex				
Female	50 (76%)	32 (72.7%)	1.171	1.0
Male	16 (24%)	12 (27.3%)		
Age ranges (y)				
<45	42 (63.6%)	5 (11.9%)	0.119	0.001*
45–59	19 (28.8%)	22 (52.4%)	9.73	
60–80	5 (7.6%)	15 (35.7%)	25.20	
MM duration (y)				
0.5–1		10 (23.8%)		
2–5		29 (69.5%)		
5–10		3 (7.1%)		

*Note*: The data is presented as total numbers, percentages, and OR with 95% confidence intervals, using the control group, female sex, and the youngest age category as reference groups. Cochran‐Armitage test for trend was specifically applied to the ordinal age variable to evaluate ordinal categories. A significant difference is indicated by the asterisk.

Abbreviations: MM, multiple myeloma; OR, odds ratio; y, years.

Age distribution differed markedly between groups: 63.6% of controls were under 45 years, compared to 11.9% of MM patients; 28.8% of controls were aged 45–59 years, versus 52.4% of MM patients; and 7.6% of controls were aged 60–80 years, compared to 35.7% of MM patients. Analysis of the association between age group and disease status revealed a significant trend. Using the <45 years category as the reference, the odds of belonging to the MM group were significantly higher in older age categories (45–59 years: OR = 9.73, 95% CI [3.2, 31.4]; 60–80 years: OR = 25.20, 95% CI [7.4, 91.0]). This increasing gradient with age was confirmed as statistically significant by a Cochran‐Armitage test for trend (*p* < 0.0001).

### 3.2 Sleep Quality Assessments

PSQI evaluation revealed that 39.4% of control volunteers exhibited good sleep quality (PSQI score 0–5) compared to 25% of MM patients. Among MM patients, 47.7% scored 6–10 points (poor sleep quality) and 27.3% scored >10 points (significant sleep disturbance), compared to 42.4% and 18.2% of controls, respectively (Table 2).

**TABLE 2:** Pittsburgh Sleep Quality Index (PSQI) scores by cutoffs.

PSQI (cutoffs)	Groups			
	Control		MM	
	*n*	%	*n*	%
Good sleep quality (0–5 points)	26	39.4	11	25
Bad sleep quality (6–10 points)	28	42.4	21	47.7
Sleep disturbance (>10 points)	12	18.2	12	27.3
Total	66	100	44	100

**FIGURE 1:**
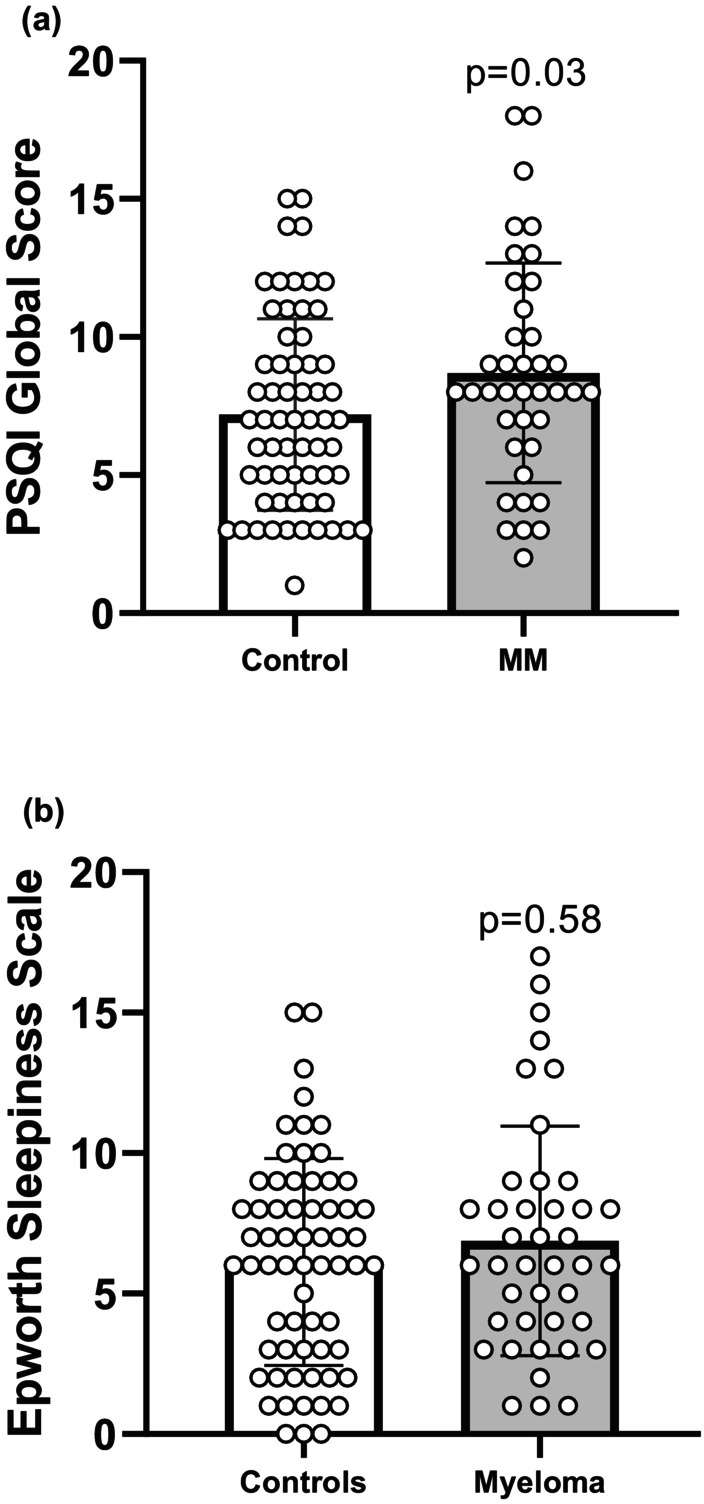
Evaluation of sleep quality and daytime sleepiness of multiple myeloma (MM) volunteers compared to a control group. (a) Pittsburgh Sleep Quality Index (PSQI) global score, control group (*n* = 64), multiple myeloma (MM) group (*n* = 46). (b) Epworth Sleepiness Scale (ESS), control group (*n* = 64), multiple myeloma (MM) group (*n* = 41). Data are presented as the mean ± standard error of the mean. Statistical analyses were performed using the Mann–Whitney *U* test for two‐group analyses.

Analysis of PSQI components demonstrated statistically significant group effects on the scores of the components: sleep latency (U = 1030.5, *p* = 0.011), sleep duration (U = 942.5, *p* = 0.0013), and use of sleep medication (U = 1192, *p* = 0.0056) (Table 3). Global PSQI scores were significantly higher in the MM group compared to controls (U = 1126, *p* = 0.034) (Figure 1a). In contrast, no significant difference in sleepiness was observed between groups as measured by the ESS (U = 1228, *p* = 0.58) (Figure 1b).

**TABLE 3:** Pittsburgh Sleep Quality Index (PSQI) scores of the groups.

Components	Groups		Values	
	Control	MM	U	*p*
Subjective sleep quality	0.92 (0.89)	1.24 (1.07)	1244	0.128
Sleep latency	0.72 (0.84)	1.14 (1.05)	1030.5	0.011*
Sleep duration	0.57 (0.74)	1.09 (0.98)	942.5	0.0013*
Habitual sleep efficiency	0.54 (0.86)	0.78 (1.19)	1294	0.558
Sleep disturbances	1.18 (0.49)	1.28 (0.54)	1420	0.373
Use of sleep medication	0.36 (0.92)	0.96 (1.33)	1192	0.0056*
Daytime dysfunction	0.88 (0.93)	1.0 (1.03)	1458	0.611

*Note*: Parameters were compared by the unpaired Mann–Whitney *U* test. All the data are presented as mean (± standard deviation). Significant differences are indicated by the asterisk.

Abbreviation: MM, multiple myeloma.

**FIGURE 2:**
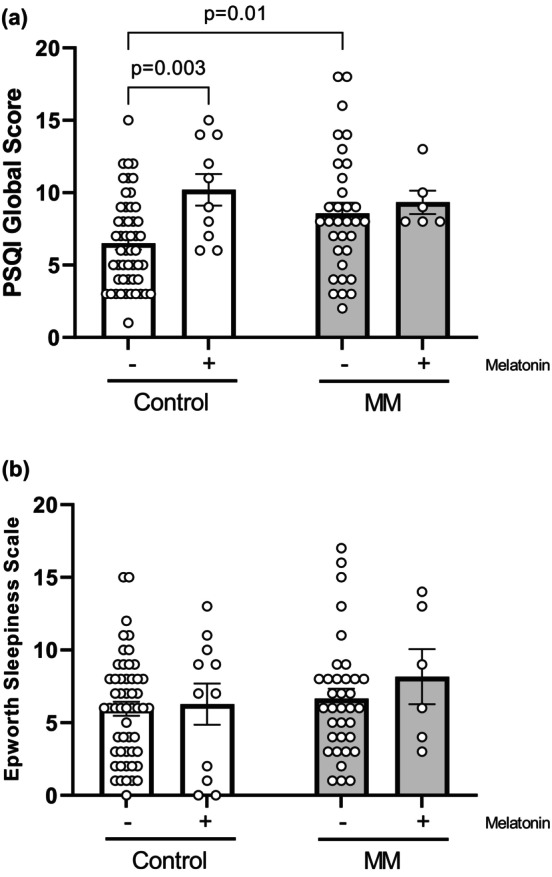
Evaluation of sleep quality and daytime sleepiness of multiple myeloma (MM) volunteers under melatonin treatment. (a) Pittsburgh Sleep Quality Index (PSQI) global score, control group melatonin (−) (*n* = 51), control group melatonin (+) (*n* = 10), multiple myeloma (MM) melatonin (−) (*n* = 33), multiple myeloma (MM) melatonin (+) (*n* = 6). (b) Epworth Sleepiness Scale (ESS), control group melatonin (−) (*n* = 53), control group melatonin (+) (*n* = 11), multiple myeloma (MM) melatonin (−) (*n* = 35), multiple myeloma (MM) melatonin (+) (*n* = 6). Data are presented as the mean ± standard error of the mean. Statistical analyses were performed using the Kruskal–Wallis test followed by Wilcoxon post hoc tests with Bonferroni correction for stratified comparisons of melatonin treatment effects.

Effects of melatonin treatment melatonin administration significantly increased global PSQI scores in the control group [control (+)] compared to untreated controls [control (−)] (*p* = 0.003) (Figure 2a). Similarly, MM patients not receiving melatonin [MM (−)] showed significantly higher PSQI scores compared to untreated controls (*p* = 0.01), with significant main effects for both group (H = 21.234, *p* < 0.001) and treatment (H = 14.963, *p* < 0.001) factors (Figure 2a). Conversely, ESS scores revealed no significant differences, with nonsignificant effects for both group (H = 2.245, *p* = 0.134) and treatment (H = 0.037, *p* = 0.847) factors (Figure 2b).

## 4 DISCUSSION

To our knowledge, this is the first observational study to describe the occurrence and severity of sleep disturbances in MM and to investigate a potential beneficial sleep effect promoted by melatonin in patients with MM. The demographic characteristics of our study participants revealed a similar gender distribution in both groups. The OR of being male was 1.17 times higher in the MM group compared to the control group, indicating no statistically significant association between sex and volunteer groups. This pattern is in line with the typical demographic profile of MM, which generally shows nearly equal gender distribution (46.9% female, 53.1% male) (Hungria et al., 2017). The age distribution showed a significant increasing gradient, wherein the odds of belonging to the MM group rose with advancing age (45–59 years: OR = 9.73, 60–80 years: OR = 25.20) compared to the control group, which closely aligns with reports of age distribution at diagnosis (Hungria et al., 2019), reflecting the higher prevalence of the disease in older adults.

Also, overall survival in MM is highly variable, ranging from months to over a decade (Rajkumar & Kumar, 2016). Our data corroborate this, showing that most MM volunteers (69.5%) were undergoing treatment for 2–5 years, while a smaller subset (7.1%) had been in treatment for over 5 years. Furthermore, most of the participants in the MM group (66.7%) were not working at the time of the survey (data not shown), a common consequence of the disease and its treatment.

Regarding the PSQI global score, we detected an overall worsening in sleep quality in the MM group that was supported by the massive identification (through the cutoffs) of bad sleep quality or sleep disturbances in 75% of the MM volunteers. A closer look at the PSQI components revealed significant worsening, in the MM group, on the scores of sleep latency, sleep duration, and use of sleep medication components. These results characterize the occurrence of sleep disruption in MM patients (through the PSQI global score) and suggest which specific sleep components may be most affected by the disease or its treatment. Sleep disturbances in hematological malignancies have previously been reported in cohorts including patients with acute myeloid leukemia, acute lymphoblastic leukemia, and non‐Hodgkin lymphoma, in which similar classifications of sleep quality were assigned based on PSQI scores (Castelli et al., 2022). Here, we contribute new data on patients with MM, adding information on sleep quality across three major groups of hematological cancers and demonstrating that sleep problems are a common condition among them. While sleep disturbances in hematological cancer patients are often considered symptoms of cancer‐related fatigue, in MM an aggravating factor is the presence of bone pain, reported in approximately 80% of cases (Shapiro et al., 2021). Additionally, all therapeutic regimens include the prolonged use of corticosteroids such as dexamethasone, which are associated with sleep dysfunction, including insomnia (Shapiro et al., 2021), corroborates with PSQI evidence recovered here.

In view of these aspects, the application of the PSQI in MM patients seems to have a good utility in assessing the presence and severity of general sleep abnormalities, encompassing the patient's perception of their sleep quality. Despite the results of sleep quality, we failed to detect increased daytime sleepiness in the MM group since ESS was no different from the control group. A mild 16.9% of MM patients scored above the clinical cutoff of 10, suggestive of excessive daytime sleepiness (EDS), compared to 11.0% of controls; however, without statistical significance.

This dissociation between sleep quality and daytime sleepiness is recognized in the literature, as they are governed by distinct physiological and behavioral mechanisms. Subjective sleep quality can be adversely affected by factors such as sleep fragmentation, micro‐arousals, and stress, even without a corresponding increase in objective sleepiness (Bonnet & Arand, 2003). Daytime sleepiness is more closely tied to severe sleep deprivation or specific disorders like sleep apnea, whereas sleep quality reflects perceived disturbances in latency, efficiency, and continuity (Banks & Dinges, 2007). Furthermore, adaptive mechanisms, such as increased slow‐wave sleep, may compensate for poor sleep quality, mitigating subjective sleepiness and explaining the observed discrepancy (Bonnet & Arand, 2003; Halász et al., 2014). Thus, our MM cohort may experience significantly impaired sleep quality without manifesting clinically relevant EDS.

The medication profile of the volunteers (data not shown) was consistent with the expectations for an MM cohort. After immunomodulators/antineoplastic agents (lenalidomide and thalidomide, used by 56% of patients), the most commonly used drug classes were cardiovascular agents (39%) and antibiotics/antivirals (38%). This pattern is anticipated, as MM patients exhibit a heightened predisposition to infections (Silva et al., 2009) and frequent comorbidities such as hypertension (49.2%) (Tietsche De Moraes Hungria et al., 2019). The comparable rates of self‐reported melatonin use in both groups (15.4% controls vs. 14.6% MM) are notable, given the significantly worse sleep quality in the MM cohort. This finding may be influenced by several factors, including its regulatory context. In Brazil, melatonin is classified as a dietary supplement by ANVISA (Agência Nacional de Vigilância Sanitária), a status that may contribute to its under‐recognition as a therapeutic option within formal oncology care. In complex treatment regimens, sleep complaints may be deprioritized or managed with prescription medications instead. Additionally, the presence of melatonin users in the control group aligns with our broad inclusion criteria, as control volunteers were not required to be free of sleep complaints. This reflects real‐world patterns in which individuals may use over‐the‐counter supplements for mild or intermittent sleep issues. It should be noted that, while the Brazilian Sleep Society recognizes melatonin's role in managing circadian rhythm disorders, evidence supporting its efficacy for insomnia remains inconsistent, despite a favorable safety profile and reported benefits in specific populations (Bacelar & Pinto Jr, 2020).

Our results demonstrate that exogenous melatonin did not alter ESS scores, indicating no significant effect on daytime sleepiness. Conversely, significant impairments were observed in the specific PSQI components of sleep latency, sleep duration, and use of sleep medications. Paradoxically, melatonin was associated with poorer global sleep quality in controls, as measured by the PSQI. Critically, however, untreated MM patients also exhibited significantly higher PSQI scores than untreated controls. This pattern strongly implicates MM itself as a direct contributor to poor sleep quality, an effect that melatonin supplementation did not ameliorate in our cohort.

It has been previously reported that melatonin decreases sleep onset latency, increases total sleep time, and improves overall sleep quality, which is fundamentally tied to circadian regulation rather than direct hypnotic properties (Ferracioli‐Oda et al., 2013). Notably, exogenous melatonin modulates the GABAergic system via MT1 receptors in the lateral hypothalamus by inhibiting hyperpolarization‐activated cyclic nucleotide‐gated ion channels and via MT2 receptors in the thalamic reticular nucleus (Huang et al., 2020; Ochoa‐Sanchez et al., 2011). This subcortical mechanism is particularly salient in cancer populations, where circadian disruption‐manifesting as clinically significant rest activity rhythm disorders constitute a primary driver of sleep–wake disturbances, independently contributing to symptom burden and impaired quality of life (Gouldthorpe & Davies, 2025). Our findings suggest that melatonin may address specific circadian‐mediated sleep complaints in MM patients without directly influencing daytime alertness, thereby leaving key aspects of cancer‐related fatigue unchanged. Therefore, it is plausible that exogenous melatonin modulated specific sleep parameters related to circadian timing and perception of sleep quality in MM patients without directly influencing their daytime alertness.

This study has some limitations that must be stated. Regarding statistical design, the use of conservative post hoc corrections (Bonferroni), while reducing the risk of Type I errors (false positives), may have increased the risk of Type II errors (false negatives), particularly in the analysis of significant Kruskal–Wallis results in the melatonin treatment analysis. This, combined with the inherent noise of self‐reported sleep data and the limited sample size, means that some real associations or treatment effects may have been undetected. Consequently, the significant findings reported are likely robust, but the analyses should be considered exploratory where multiple comparisons were involved, and the results require validation in larger, confirmatory studies. Also, the PSQI global score focuses on sleep habits and may not capture specific sleep disorders prevalent in this population, such as restless legs syndrome or rapid eye movement (REM) sleep behavior disorder. Finally, variability in melatonin pharmacokinetics and administration timing may have influenced the results and could not be controlled in this study.

Our findings demonstrate that while exogenous melatonin did not improve daytime sleepiness in MM patients, it significantly influenced PSQI parameters. This aligns with the growing recognition of the circadian system's role in regulating key biological processes, including the mechanisms of action and toxicity of anticancer drugs (Biso et al., 2025; Ozturk et al., 2017). Consequently, cancer chronotherapy is gaining attention for its potential to enhance the efficacy and tolerability of treatments. Despite the limitations of our data regarding melatonin treatment, the literature shows that it holds promise not only as a chronobiotic agent for ameliorating sleep disturbances but also for its potential oncostatic effects, as evidenced by promising preclinical studies across various cancers (Li et al., 2017; Mogavero et al., 2021). Therefore, we propose that the integration of melatonin into the management of MM represents a compelling strategy for a multifaceted, patient‐centered approach, targeting the pervasive issue of sleep disruption while potentially conferring broader therapeutic benefits.

## 5 CONCLUSION

In summary, this study provides the first systematic characterization of sleep disturbances in MM, establishing that poor sleep quality is a prevalent and inherent feature of the disease, primarily affecting sleep latency, duration, and medication use. While exogenous melatonin influenced specific circadian‐mediated sleep parameters, it did not improve global sleep quality in MM patients or alleviate daytime sleepiness, highlighting a dissociation between these domains. Our findings underscore the critical need to recognize and routinely assess sleep disturbances in MM management. Future research should prioritize controlled chronotherapy trials with standardized melatonin protocols to further establish its potential utility for improving sleep in MM patients.

## AUTHOR CONTRIBUTIONS

Conceptualization: M.M.S.L. Formal analysis: M.F., E.M.A., L.S.R., D. P. B., and M. M. S. L.; Investigation: M.F., E.M.A., L.S.R., D.P.B., and M.M.S.L. Methodology: M.F., E.M.A., L.S.R., D. P. B., and M. M. S. L. Roles/Writing—original draft: M.M.S.L. Writing—review and editing: M.F., E.M.A., L.S.R., D.P.B., and M.M.S.L.; Funding acquisition: D.P.B. and M.M.S.L.

## FUNDING INFORMATION

This study was supported by the FINEP (Grant # 1648/22) and Conselho Nacional de Desenvolvimento Científico e Tecnológico (CNPq), Brazil, Grant #444884/2024‐6—CNPq/MCTI/FNDCT N° 22/2024—Programa Conhecimento Brasil to MMSL. DPB and MMSL are recipients of CNPq fellowships (Grant #312982/2025‐9 for DPB, Grant #306432/2022‐6 for MMSL).

## CONFLICT OF INTEREST STATEMENT

The authors declare no conflict of interest.

## Data Availability

The authors declare that the data will be available upon reasonable request.
